# Preventable contributors to the neonatal healthcare-associated infections: a uni-center analytical study from South India.

**DOI:** 10.12688/f1000research.111101.1

**Published:** 2022-04-25

**Authors:** Usha Rani, Leslie E. Lewis, Kiran Chawla, Anup Naha

**Affiliations:** 1 Department of Social and Health Innovation, Prasanna School of Public Health, Manipal academy of Higher Education, Manipal, Karnataka, 576104, India; 2 Department of Pediatrics, Kasturba Medical College, Manipal Academy of Higher Education, Manipal, Karnataka, 576104, India; 3 Department of Microbiology, Kasturba Medical College, Manipal Academy of Higher Education, Manipal, Karnataka, 576104, India; 4 Department of Pharmaceutics, Manipal College of Pharmaceutical Sciences, Manipal Academy of Higher Education, Manipal, Karnataka, 576104, India

**Keywords:** Cross infection, neonate, healthcare, prevention

## Abstract

Background: Globally, neonatal healthcare-associated infections (HAIs) are known to cause high mortality. HAIs is a preventable condition related to the healthcare environment. The current study explored the contributors to neonatal HAIs in one of the largest tertiary care referral hospitals in South India.

Methods: Neonates from December 2016 to June 2018 were observed for the occurrence of healthcare-associated infections and compared with the matched control group. Various observations on neonatal demography, maternal contributors, and medical procedures were made and recorded to explore and analyse the contributors to neonatal HAIs. Univariate and multivariate analysis was carried out to find the contributors. The Odds ratio with 95% CI was also computed and reported.

Results: Bloodstream infection (83%) was prevalent among neonates; the maternal contributor was only preterm labor (Odds ratio of 11.93; 95% CI; 6.47-21.98; p<.05) to acquire HAIs. On univariate analysis, mechanical ventilation for > 3days duration, NIV for > five days, and PICC line insertion procedure were significant (p<0.05) contributors to neonatal HAIs. IV cannulation for more than three times in four consecutive days was found in 100(85%) neonates considered being associated with neonatal HAIs. On multivariate analysis, NIV, PICC line, preterm labor, and low birth weight were significant (p<0.05) contributors to neonatal HAIs.

Conclusion: The increased duration of invasive and non-invasive therapeutic devices and catheters contributes to neonatal HAIs. Neonates are acquiring bloodstream infections; low birth weight (LBW) neonates are more susceptible to acquiring HAIs.

## Introduction

Reports from UNICEF and the World Bank 2018 showed a reduction in Neonatal Mortality Rate (NMR) among all the participating countries. India’s NMR is 23 as per 2017 report. ^1^^,^^2^ Each year nearly 0.748 million newborn deaths occur in India, contributing to 26% or 1/3rd of the world’s neonatal death. As per WHO, in developing countries like South East Asia, HAIs are responsible for nearly 50% of mortality in Neonatal Intensive Care Unit (NICU). ^3^ The rate of healthcare-associated infections (HAIs) reported by the World Health Organization (WHO) in South Asian countries ranges from 11.3-23.6%. ^4^

According to Liu *et al.*, dominating causes of neonatal deaths are preterm (35%), birth asphyxia (20%), pneumonia (16%), sepsis (15%), and other causes (<10% each group), these are also the primary reasons for ICU admission and development of HAIs. ^5^ Indian studies have reported bloodstream HAIs, Ocular infection (Healthcare-associated conjunctivitis), urinary tract infection, and skin infection among neonates. ^6^^–^^11^

Newborns needing critical care and support get hospitalized in Neonatal Intensive care units (NICUs) are at increased risk of acquiring Healthcare-associated infections (HAIs). ^12^ HAIs have a detrimental effect on the recovery, length of stay in NICUs, and immune system acting as a vicious cycle. ^13^

Neonatal HAIs is one of the preventable leading cause for neonatal mortality. Developed countries are putting efforts to identify the risk factors to neonatal HAIs and its preventable measures. ^14^^,^^15^ The contributing factors to neonatal HAIs in India are unexplored among neonates. ^16^ The rate of HAIs and their related mortality and morbidity is also not explored in Indian literature. To contain the spread of microorganism, domestic preventable contributors to neonatal HAIs needs to be explored. The objective of the study was to identify the contributors to neonatal HAIs in one study centre.

## Methods

The study collated and analysed the contributing factors from December 2016 - June 2018. The study was conducted in one of the largest tertiary care teaching referral hospital in south India. Manipal Academy of Higher Education (MAHE) ethics committee provided Institutional Ethics Committee (IEC) approval was taken, approval ID: MUEC/014/2016-17. The study setting is equipped with a 30-bedded level III Neonatal Intensive Care Unit (NICU) with mechanical ventilation, indwelling catheters, intravenous fluid, phototherapy, and an advanced monitoring facility.

ICU hospitalized neonates were followed to development of neonatal HAIs. A case-control study design was adopted with controls matching the gestational age to identify maternal and neonatal direct contributors. The neonates were defined from the day of birth ‘0’ days to ‘28’ days; however, each day of life was counted from 24 hours of birth, not at midnight.

In order to compute the sample size, the probability of HAIs in current settings was obtained from a pilot study data of the same study setting and found to be 8 per 100 admissions >48 hrs. We assumed the probability of acquiring neonatal HAIs (p) as 0.08 and the probability of not acquiring neonatal HAIs (q) as 1-p = 0.92. With a 95% confidence interval using estimation of single proportion sample size calculation method d (accepted error) = 0.05. Sample size computation was made as below: $s=Z^{2}\frac{\mathrm{pq}}{d^{2}}$ = 113 neonates with HAIs for infinite population and 106 for the finite population. We assumed 10% missed events; hence another 12 cases were added in the sample pool leading to 118 samples of neonates acquiring HAIs with similar numbers in the control group.

The case detection and confirmation were carried out for any newborn admission to NICU >48 hrs using a combination of CDC and WHO recommended clinical findings and diagnostic findings, where the presence of at least any two variables of each category confirms the case label as HAIs. ^17^^,^^18^ The following clinical and diagnostic criteria were used to determine the neonate acquiring HAIs. The presence of any two clinical and diagnostic tests was considered as the case of positive neonatal HAIs ( Table 1). The Case Record Form (CRF) was prepared keeping objectivity as priority to clearly discriminate between neonate acquiring HAIs versus other sick babies. The CRF was pilot tested for construct, content and criterion validity before the commencement of the study.

**Table 1.** Criteria determining the presence of neonatal HAIs.

Clinical Criteria, at least two of the following ^17^^,^^18^	
Admission >48 hours in ICU	Acute bradycardia <30 bpm from baseline
The acute febrile episode, temperature >38°C	Increase heart rate >180 bpm
Hypothermia, temperature <36°C	Increase respiratory rate >40 bpm
Acute apneic episode >20 second	Lethargy
Localizing neurologic signs	Paradoxical pulse
Tenderness/inflammation of the site of catheter/device insertion	Sternal instability/purulent drainage from the mediastinal area
Feeding intolerance /sudden vomiting/indigestion of enteral feed	Sudden diarrhoea: passing watery loose stool >10 times in 24 hours
Meningeal signs/cranial nerve signs	Nasal discharge/purulent exudate in the throat
A sudden increase in sputum/endotracheal aspirate or change in colour of sputum/endotracheal aspirate from white to yellow/green or consistency thin watery to sticky/thick	

Diagnostic Criteria, at least two of the following	
Leukocytosis > 12000/mm ^3^/Leukopenia <5000/mm ^3^	Immature/total neutrophil ratio >0.2
Thrombocytopenia < 100,000/mm ^3^	Positive culture
Elevated C-Reactive protein (CRP) >10 mg/dl	Organisms are seen on Gram stain of CSF (Cerebrospinal fluid)
Decreased glucose in CSF	Neutropenia < 1800/mm ^3^
Imaging test evidence suggestive of infection	Procalcitonin > 0.5 ng/ml

Neonates admitted from other hospitals with sepsis and maternal infections like UTI/Chorioamnionitis/Pneumonia were excluded from the study. Each morning of a working day the duty clinician as well as the nurse in charge was enquired for any probable case of HAIs by the researcher. If it was a holiday or weekend the next working day the enquiry was raised on suspected case of neonatal HAIs who was in NICU since last 48 hours. Only infections originating at the study site after 48 hours of admission were identified and included in the study as per the clinician ascertainment and the diagnostic criteria determining HAIs. The data on these neonates were obtained from medical records.

Bed occupancy days, admission and discharge in total per month, and further inborn and outborn categorization is reported in numbers per month. The rate of HAIs was calculated per 100 neonatal admissions for >48 hours.

Rate of HAIs per 100 neonates admitted for >48 hours = (# of new HAIs/# of all admissions in respective month >48 hours) × 100

Bacteraemia rate was calculated by dividing the number of new cases by the total number of bed occupancy days and multiplying it by 1000.

Bacteraemia rate per 1000 days = (#of HAIs per month/Total number of Bed Occupancy days in the respective month) × 1000

A Chi-square test was performed on the birthing place and HAIs to find any statistical significance among two variables where the level of significance was fixed at <0.05.

The variables were entered in Microsoft Excel sheet within 48 hours of data collection and the neonate was followed till its outcome as improved/succumbed/discharge against medical advice. If the any variable input was missed the clinician and nurse care giver was asked to provide the relevant information on missed data. Missed data was manually filled post completion of the enquiry. The descriptive variables were reported using mean and standard deviation when normally distributed. Median with interquartile range was reported for skewed data. The frequency and percentage table were used for the nominal and categorical variables. Boxplot was used for skewed data to identify and report the outliers, however, further analysis with or without outliers was not performed.

### Identifying the maternal contributors causing neonatal HAIs

Maternal risk factors like mode of delivery, premature rupture of membrane (PROM), maternal peripartum infection, preterm labor, foul vaginal discharge, maternal urinary tract infection, intrapartum fever >38°C, and uterine tenderness was recorded and analyzed for their role in HAIs. A case record form was used, and details on maternal history were obtained from the neonatal record file at the time of admission.

### Identifying the neonatal contributors causing neonatal HAIs

Neonatal healthcare-associated infections have been related to many variables, the patients’ data related to demography like gestational age, birth weight, mode of delivery and congenital deformity, gender, date of birth, date of admission, date of discharge/death/DAMA (Discharge Against Medical Advice) etc. were captured and analyzed.

Medical invasive interventions, number of intravascular lines, duration of invasive and mechanical ventilation, central line, a peripherally inserted central catheter (PICC line), medication delivery, and any other invasive procedure record were captured. Medical non-invasive interventions like duration of non-invasive mechanical ventilation, phototherapy, and routine care were recorded.

The score for neonate acute physiology (SNAP II) score for each neonate in their first 24 hours of admission was calculated and reported. Clinical diagnosis and patients’ vitals were recorded to identify cases of HAIs.

Clinicians’ ordered diagnostic biochemistry laboratory investigations, and the result of microbiological test reports in the proforma were recorded, which helped identify the cases with HAIs.

A record on the number of catheters, cannula/tubing attached, and the procedures carried on to the neonate was captured. Later retrospectively, the records were screened to find any invasive or non-invasive procedure, the medical treatment, the feeding schedule and the type of feeding given, the number of times the catheter site was touched, the number of invasive catheters, and the duration of each catheter in the body and any sign or symptom of infection was recorded. This proforma was filled within 48 hours of diagnosing HAIs in neonates. The controls of 1:1 were taken from the medical record of the neonates matching the gestational age. The recorded data was captured, and observations on the control could not be carried out.

The descriptive variables were reported using mean and standard deviation when normally distributed. Median with interquartile range was reported for skewed data. The frequency and percentage table were used for the nominal and categorical variables. A cluster bar diagram was used to summarise the categorical variables. Chi-square test was used with a level of significance at <0.05 for analyzing maternal and neonatal contributors for HAIs. The Odds ratio for demographic variables and the use of devices to ascertain HAIs was determined. Analysis of the odds of vascular catheter insertion for more than three times to HAIs was not ascertained due to insufficient controls data.

Univariate logistic regression followed by Backward Wald to identify the independent coefficient of each covariate was carried out using IBM SPSS Statistics for Windows, version 26 (IBM Corp., Armonk, N.Y., USA). Multivariate analysis of the variables found to be significant in univariate analysis was carried out to find the adjusted contributors to HAIs.

Clinical Trial Registry India (CTRI) registration was done before starting the project, and the confirmation ID was: CTRI/2017/08/009538.

## Results

### Identifying the prevalence of neonatal HAIs

The total number of admissions to NICU was 2278 neonates, with 1223 neonates hospitalized for >48 hours in nineteen months duration. Neonates hospitalized for less than 48 hours with outcome or with infection were excluded ( Table 2). The rate of HAIs was 9.6±4.1 per 100 admissions, and the bacteremia rate was 5.2±1.6 per 1000 days ( Table 2).

**Table 2.** Demographic contributors of the neonates in NICU.

Demographic variable	Sub variable	Cases with HAIs n=118 (%)	Controls without HAIs n=118 (%)	Total n (%)
Place of birth	Inborn	72 (51)	70 (49)	142 (100)
	Outborn	46 (49)	48 (51)	94 (100)
Outcome	Mortality	29 (60)	19 (40)	48 (100)
	Improved	85 (46)	99 (54)	184 (100)
Gender	Male	70 (48)	77 (52)	147 (100)
	Female	48 (54)	41 (46)	89 (100)
Gestational age *	Extreme Preterm <28 week	14 (50)	14 (50)	28 (100)
	Very preterm 28-<32 week	37 (50)	37 (50)	74 (100)
	Moderate to late preterm 32-<37 week	46 (50)	46 (50)	92 (100)
	Term >37 week	21 (50)	21 (50)	42 (100)
Birth weight	Extreme low birth weight	27 (56)	21 (44)	48 (100)
	Very low birth weight	41 (51)	39 (49)	80 (100)
	Low birth weight	27 (36)	24 (64)	51 (100)
	Normal birth weight	23 (48)	34 (52)	57 (19)

* Gestational age was taken for matching.

The median length of stay for all the admissions to NICU was 10 days (IQR=8.9-11), and for cases with HAIs, it was 30 days (IQR=16-45), we did not find any correlation of length of stay with any specific microorganism as found in other studies. ^19^ The length of stay for cases with HAIs ranged from four days to 147 days. Neonatal stay in NICU for >48 hours was found in 45% of all admissions to NICU.

The SNAP II score ranged from 0 to 94, where 79% of neonates had a score of ‘0’; 5% had a score of 5, and there were 11% neonates with a score of >5 within 24 hours of admission. Among the 5% of neonate in case group and all the patients in control group; the SNAP II score could not be calculated due to the unavailability of the required data. The SNAP II score was computed to describe the severity of sickness among the neonate on admission to NICU and was not considered as contributor to HAIs.

There were four neonates with congenital issues where two had intrauterine growth retardation (IUGR), one with hypoxic-ischemic encephalopathy, and one had hypoglycemia and seizures. Since the data was captured from medical records and were also verified by physician there was no variable considered to be contributing to HAIs had missing data.

Mothers of the neonate in the very preterm category had experienced more premature rupture of the membrane (PROM) (57%) as compared to others. These neonates had a better outcome (72%) and were improved with treatment even though they acquired HAIs. However, any neonate born to a mother with a history of PROM >18 hours was excluded from the study.

Mothers of moderate to late preterm neonates acquiring HAIs experienced preterm labor (44%) more compared to any other group and was found to be statistically significant at p=0.000. There was 24 (27%) mortality among neonates with HAIs born to mothers with preterm labor, whereas 63 (69%) neonates had recovered. There was no statistically significant association (p=0.2504) between maternal contributors and the neonate’s outcome (mortality vs. improved).

Preterm labour was noted among ninety-one mothers of neonate acquiring HAIs but the first culture sample among all these neonates did not grow any microorganisms. No mother was identified with intrapartum fever >38°C or uterine tenderness or Maternal leukocytosis >15000/mm ^3^. We found preterm labor with an Odds ratio of 11.93 (95% CI; 6.47-21.98; p<0.0005) contributed to the development of HAIs in univariate analysis,

### Identifying the neonatal contributors causing neonatal HAIs

Eighty-three (70%) neonates acquiring HAIs were delivered through cesarean section (C-Section), similar to studies reported from India. ^19^ Most of the neonates were improved and discharged (71%) from NICU; however, mortality (25%) and discharge against medical advice (4%) outcome was recorded, in control group there was 19 (16%) neonates died and 99 (84%) neonates improved ( Table 2). The overall mortality during the study period in the entire NICU was 5% among all neonatal admissions, whereas there was 25% mortality among the neonates acquiring HAIs, contributing to nearly 1/4 ^th^ mortality (24%) among all the admissions to NICU. The Odds of mortality were found to be insignificant (Odds ratio=1.777; 95% CI=0.93-3.39; p=0.0814) among cases with HAIs compared to non-HAIs cases.

The male gender (59%) acquired HAIs more than the female gender (41%). Moderate to late preterm neonates (38%) acquired HAIs more compared to the rest of the categories. Mean gestational age was 32±4.3; the gestational age showed near-normal distribution among neonates with HAIs.

On univariate analysis, we did not find any birth weight category as a contributor to neonatal HAIs both the Odds ratio and chi-square test were insignificant to HAIs; ELBW (p=0.339), VLBW (p=0.7833), LBW (p=0.6353), NBW (p=0.09).

Most of the neonates acquiring HAIs were born through C-section (71%), were VLBW with a median birth weight of 1370 (IQR=530, 3860) grams. The majority of the neonate acquiring HAIs were male gender (59.3%) and were moderate to late preterm (39%) with VLBW (35%). Further analysis between gender and birthweight showed that the male gender with VLBW (19%) was more prone to HAIs than females in a similar birth weight category (16%) ( Table 2).

**Figure 1.**
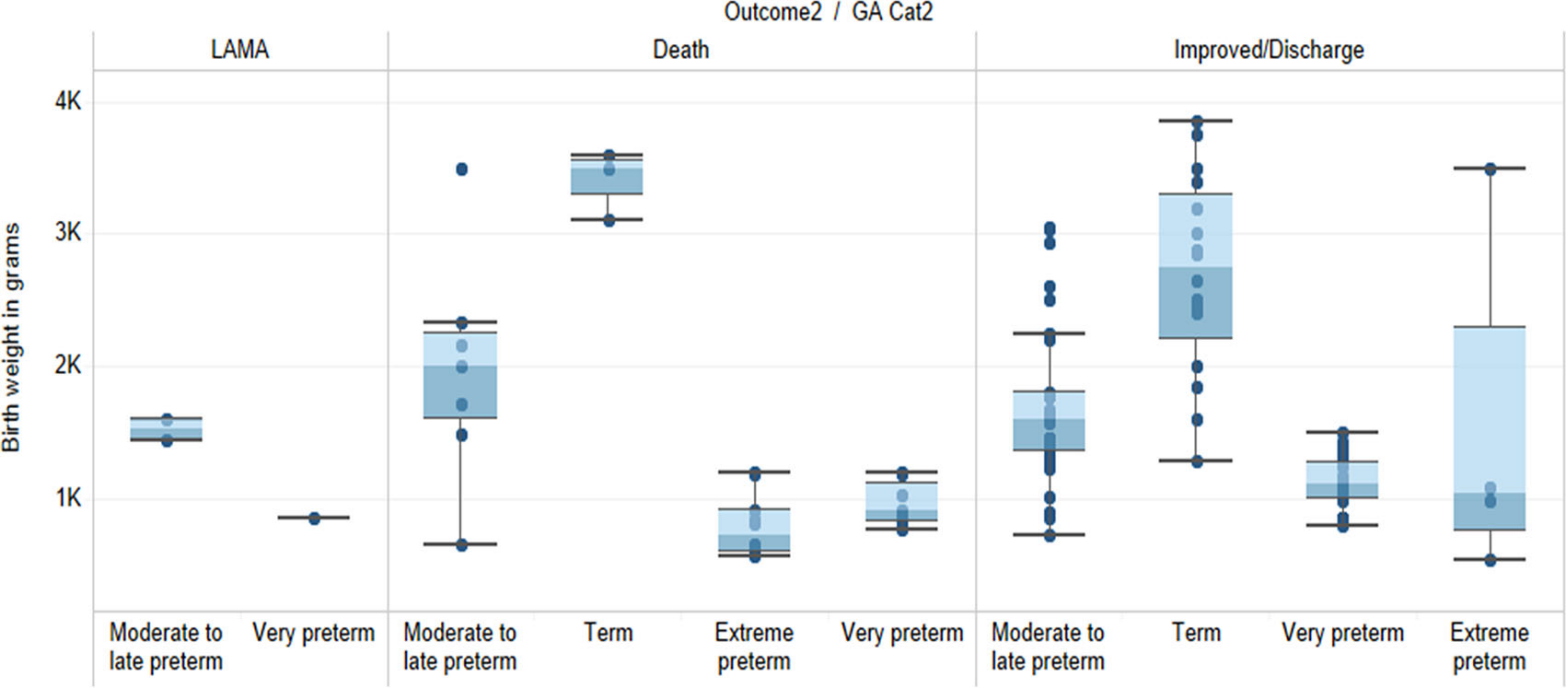
Outcome, gestational age and birth weight distribution of the neonates with HAIs (1K = 1000 grams).

Overall extreme preterm neonates (38%) had detrimental outcome (mortality) as compared to the rest of the gestational age group. Neonate with moderate to late preterm with LBW acquiring HAIs had higher mortality than any other gestational age or birth weight. Neonates had birth weight ranging from 520 g to 3850 g showed marked improvement in health conditions over time and were discharged ( Figure 1).

### Use of devices/procedures as a contributor to neonatal HAIs

Invasive and non-invasive types of assisted ventilation were provided to 82% of the neonates who acquired HAIs, and 56% of neonates with HAIs were provided invasive mechanical ventilation for <5 days. Maximum neonates acquiring HAIs had <3 days of invasive mechanical ventilation and <6 days of non-invasive ventilation ( Table 3).

**Table 3.** Univariate analysis for the contributors to neonatal HAIs.

S. No	Contributors		-2log likelihood	Wald	S.E.	Odds ratio	95% CI	p-value	Chi-square	p-value
1	Birth weight (g)	<1000	324.590 ^a^	2.456	.402	1.378	.724, 2.593	>0.05	2.576	>0.05
		1000-<1500		1.159	.353	1.078	.629, 1.849	>0.05		
		1500-<2500		.733	.386	1.162	.624, 2.162	>0.05		
		>2500		2.544				>0.05		
2	MV		307.874 ^a^	18.169	.289	2.116	1.244, 3.597	<0.05	7.798	<0.05
3	NIV		299.493 ^a^	26.026	.277	3.07	1.80, 5.23	<0.05	17.48	<0.05
4	PICC Line		318.920 ^a^	7.576	.385	5.53	2.19, 13.99	<0.05	15.53	<0.05
5	Umbilical Line		320.400 ^a^	6.519	.316	.572	.304, 1.07	>0.05	3.026	>0.05

Mechanical ventilation > 3days on the chi-square test was significant at p<0.05 to neonatal HAIs. The Odds of neonates on mechanical ventilation were at 2.1 times higher risk (Odds ration=2.11; 95% CI=1.24-3.59; p=0.0056) of acquiring HAIs compared to those who were not mechanically ventilated ( Table 3).

Before diagnosing HAIs, apnoea and bradycardia were observed in 55 (47%) and 75 (64%) neonates. Both apnoea and bradycardia were observed in 41 (35%) neonates and an Irregular heart rate with apnoea was noted among 26 (22%) neonates that required immediate respiratory support, and later in two to three days’ time, neonates were identified as acquiring HAIs.

The odds of acquiring HAIs due to utilization of NIV was three times higher (Odds ratio=3.07; 95% CI=1.80-5.23; p<0.0001) compared to non-utilization of NIV. In 69 (58%) neonates, Feed intolerance was noted before acquiring HAIs.

There were 100 (85%) neonates who received >3 times IV cannulation procedure and developed HAIs within 72-96 hrs of the procedure. There were 35 (30%) neonates having either peripherally inserted central catheter or umbilical catheter who acquired HAIs when these lines were in situ. Such neonates’ blood samples were sent within 72-96 hrs to the microbiology lab for culture and sepsis screen. The presence of a PICC line on univariate analysis was a significant (p<0.0003) contributor to neonatal HAIs. PICC line presence carries an Odds of 5.5 times higher risk (Odds ratio=5.538; 95% CI=2.192-13.99; p≤0.001) to pose HAIs. In contrast, the presence of an umbilical catheter did not pose threat to cause HAIs ( Table 4). The Chi-square (chi-square statistic is 15.5361) test was also significant for the PICC line (p=0.000081) but not for umbilical line insertion for HAIs ( Table 4).

**Table 4.** Devices & catheters utilization duration among neonates with HAIs.

Procedure	Type	Variable	Observations n (%)	Statistical significance
Assisted Ventilation	Mechanical ventilation (MV)	<3 days	43 (36%)	p<0.05 overall for MV p<0.05 * for MV > 3days
		3-4 days	24 (20%)	
		5-6 days	18 (15%)	
		> 6 days	21 (18%)	
		No MV	12 (10%)	
	Non-invasive mechanical ventilation (NIV)	1-5 days	45 (38%)	p<0.001 * for NIV duration > 5 days
		6-10 days	27 (23%)	
		11-15 days	17 (14%)	
		>16 days	8 (7%)	
		No NIV	21 (18%)	
	Both types of mechanical ventilation		97 (82%)	-
	No assisted ventilation		11 (9%)	-
Vascular procedures	Umbilical catheter insertion <4 days but >1 day		24 (20%)	p>0.05 *
	PICC line insertion < 4 days but >1 day		34 (29%)	p<0.001 *
	>3 times IV cannulation procedure <4 days but >1 day		100 (85%)	-

* Chi-square test was performed with 95% CI and level of significance at p<0.05.

### Peripheral vascular line as a contributor to HAIs

Change of IV cannula for more than three times in less than four days was observed in 100 (85%) neonates who acquired HAIs later on. Those neonates who had confirmed BSI 98 (83%) among them 85 (87%) neonates had IV cannulation changed for > three times in the last four days before the occurrence of BSI. There were 33 (28%) neonates who had both PICC lines and had a change of IV cannulation > three times before the development of HAIs. We could not carry out further analysis due to the lack of recorded data on IV line insertion in the control group.

Multivariate analysis showed NIV (p=0.000; 95%CI), PICC line (p=0.005; 95%CI), preterm labour (p=0.000; 95%CI) and LBW (p<0.05; 95%CI) as contributors to neonatal HAIs. Odds of neonate on NIV posed a 2.1 times higher risk (Odds ratio 2.133; 95% CI=1.097-4.149) to the development of HAIs. The presence of a PICC line carries 6.5 times higher risk (Odds ratio 6.595; 95% CI=2.104-20.665) to HAIs. Preterm labour (Odds ratio 14.911; 95% CI=6.514-34.134) and very low birth weight (VLBW) (Odds ratio 3.371; 95% CI=1.169-9.717) pose 14.9 and 3.4 times higher risk respectively to the occurrence of neonatal HAIs. However, mechanical ventilation, umbilical catheter, PROM, ELBW, LBW, and normal birth weight (NBW) do not pose a statistically significant risk to acquiring HAIs found on multivariate analysis.

## Discussion

As per a systematic review burden of HAIs ranged from 3.6 to 11.6 per 100 neonatal admissions, whereas in low-middle income countries, it ranges from five to 19 per 100 neonatal admissions. ^4^ The incidence density of HAIs in the USA and Europe ranges from 13.0 to 20.3 incidence per 1000 hospital bed days. A multicentre study from Canada and another one from Germany reported the rate of HAIs as 23.5% and 12.3% among 100 neonatal admissions. ^20^^,^^21^

In Brazil rate of HAIs ranged from 12.3 % in NBW neonates and up to 51.9% in very low to extreme low birth weight neonates (ELBW). The overall incidence density bacteremia rate was 24.9 per 1,000 hospital bed days. ^22^^,^^23^ Another large study from Germany has reported BSI incidence as 6.5 per 1000 hospital bed days. ^24^ In South Asia, the incidence density of neonatal HAIs reportedly is 9.8 per 1000 live births. ^25^

The rate of HAIs and the bacteremia rate in the current study was similar to a study conducted in Italy where the rate of HAIs was 9%, but incidence density was lower 3.5 per 1000 hospital bed days compared to the current study. ^26^

Neonates acquiring HAIs and delivered to mother with PROM had no different outcome, however, is considered as a risk for HAIs ^27^^,^^28^ and were born to very preterm and moderate to late gestational age. Maternal peripartum infection, ^29^ UTI and leaking per vaginal had no different outcomes among neonates acquiring HAIs. However, the number of cases with HAIs were very few to analyse.

Mothers with preterm labor observed high neonatal mortality (35%), India witnesses the highest preterm deliveries in the globe and 50% of neonatal death occurs in preterm (<37 weeks gestation). ^30^ We observed high mortality among moderate to late preterm neonates (34%) born to mothers with preterm labor, probably due to the high number of neonates in this category acquiring HAIs.

Neonates acquiring HAIs and delivered to mother with PROM had no different outcome, however, is considered as a risk for HAIs ^27^^,^^28^ and were born to very preterm and moderate to late gestational age. Maternal peripartum infection, ^29^ UTI and leaking per vaginal had no different outcomes among neonates acquiring HAIs. However, the number of cases with HAIs were very few to analyse.

Mothers with preterm labor observed high neonatal mortality (35%), India witnesses the highest preterm deliveries in the globe and 50% of neonatal death occurs in preterm (<37 weeks gestation). ^30^ We observed high mortality among moderate to late preterm neonates (34%) born to mothers with preterm labor, probably due to the high number of neonates in this category acquiring HAIs.

Another known contributor to neonatal HAIs is mothers unpasteurized breast milk that was contaminated with MRSA leading to neonatal HAIs in one of the studies, however, we did not find any such contributors during our study. ^31^

Preterm labour, that was significantly associated with neonatal HAIs alone may not be able to contribute to HAIs, it can lead to lower gestational age and LBW delivery of the neonate that might contribute to a more significant extent on acquiring HAIs.

Basu *et al.* ^32^ found no significant association between the occurrence of HAIs and mode of delivery but contradictory to another study published from North-eastern India where they found a significant association between vaginal delivery (p=0.002) and occurrence of HAIs, ^33^ we did not find any association with mode of delivery.

The mortality of near 25% was similar to the study by Bammigatt *et al.* ^34^ where they also reported 24% mortality with no statistically significant (p>0.05) among cases with HAIs.

HAIs are always known to cause higher related mortality and cause of concern among all the risk factors for mortality. ^12^^,^^35^ Neonatal mortality among developing nations due to HAIs ranges from 4% to 56% of all causes of mortality during the neonatal period, ^36^ and our study found 24% of mortality was due to HAIs.

We found a predominance of male (59.3%) gender in neonatal HAIs in similar studies from the same region where they found a male predominance of 62.3%, and in another study, it was found to be 1.3:1 for the male to female ratio in acquiring HAIs in India. ^8^^,^^28^ A systematic review on neonatal sepsis found that the male gender (OR: 1.3, 95% CI: 1.02, 1.68) is a risk factor. ^37^

Those born preterms had a 92% increase in the risk of getting HAIs compared to other gestational ages, as reported in studies from developed and developing nations where preterm birth is a risk of acquiring HAIs. ^25^^,^^38^^,^^39^ The number of moderate to late preterm neonates who acquired HAIs was more than other gestational age groups, and the reason could be the higher number of admission for 48 hours and beyond to NICU of moderate to late preterm neonates compared to other gestational age. ^40^^,^^41^ The majority of neonates in this category was admitted for respiratory distress syndrome.

The neonatal HAIs among neonates with birth weight >1500 grams (62%) was reported as significant from north India. ^42^ Another large study from Germany has reported a high prevalence of HAIs among VLBW neonates. ^24^

LBW neonates are more susceptible to neonatal HAIs than NBW categories, ^26^ but our findings were in contrast. In our study, most of the neonates were in the VLBW category (35%); there was an equal proportion of birth weight distribution among ELBW (23%) and LBW (23%); however, the NBW neonates (19%) were very few in numbers.

VLBW and prematurity were identified as factors contributing to neonatal HAIs in a study from western India. ^43^ VLBW with very preterm neonates and moderate to low birth weight with moderate to late preterm were found susceptible to HAIs in our study, similar to a study from Northern India that reported LBW as a risk factor to neonatal HAIs. ^44^

However, in this study, we found only three VAP cases, and the majority were bloodstream infections still, the presence of mechanical ventilation was identified as a contributor to HAIs. Mechanical ventilation with intubation was found to be a risk to neonates causing HAIs. ^45^^,^^46^ The neonates exposed to non-invasive ventilation had an insignificant risk of acquiring HAIs, similar to other studies showing that the increased duration of NIV utilization poses a higher risk of acquiring HAIs. ^24^^,^^47^

In this study, 97 (82%) of the neonates were kept nil per oral (NPO) before developing HAIs that could have been a reason for lower immunity and infection as reported in the literature. ^48^^,^^49^

The placement of the umbilical catheter line did not show significant risk to neonates for HAIs in contrast to other studies where the author found an association in umbilical catheter insertion to HAIs. ^40^^,^^50^ We did not find any study evaluating association with the cannulation procedure in less than four days to greater than one day to the occurrence of HAIs.

We found a frequent change of IV cannula (for > 3 times) leading to multiple pricks in the skin and use of mechanical ventilation (< 3 days) was found frequent among neonates acquiring HAIs. However, further analysis could not be carried out. A study by M. Takrouri *et al.* suggests changing the IV cannula every 48 hours to prevent colonization and further infection to the patient, ^51^ but our study finds a frequent change in IV cannula poses a higher risk to neonatal HAIs. Although umbilical catheters had no significant association with HAIs, changing the central venous catheter/umbilical catheter every 10 days or less to prevent HAIs may have a better outcome. ^51^ Different therapeutic procedures, both invasive and non-invasive, significantly contribute to neonatal HAIs as per the large multicentre study of the German Neonatal Network. ^52^ To curb the development of HAIs, it is necessary to reduce the number of procedures, duration of invasive and non-invasive procedures, especially to neonates born due to premature labor and very low birth, as they pose a higher risk of acquiring HAIs.

This study had a few limitations as data collection was carried out in one study centre. In order to identify potential case of HAIs the researcher was dependent on the discretion of the duty physician and nurse. Physician initiated studies and in closed ICU would have provided further insight to the outcome. It would have been better to have cohort study rather case-control study design keeping track of all the patients irrespective of the underlying disease or HAIs. The record on number of IV site pricks and reason to remove existing IV line for all the neonate was not available and hence further analysis on this potential factor could not be carried out. Maximum data was captured from medical records of the neonate rather prospectively collected. There could be miss cases of HAIs due to selection bias created by the study data collection method that could affect the results of the study. There was no practice on collection of swab samples from various parts of ICU except on two occasions, where there was suspected outbreak of two microorganism during the study. During these two occasions identified source was from the cradle of the neonate and washbasin of NICU. Probability of missing source of infections cannot be ignored while interpreting the findings. There are many other cofactors that could be contributor to neonatal HAIs like hand hygiene of healthcare workers, ^53^ this was analysed and reported elsewhere but its establishment with cases of HAIs was not ascertained. There are other factors that were identified as contributor to neonatal HAIs elsewhere like ELBW, MV, umbilical catheter, were not found as contributor in this study setting.

As this was a uni-centre study, the results cannot be generalized to the whole population. The explored factors are similar to the published literature but some new factors that were identified and highlighted need further exploration to determine as the contributor to neonatal HAIs. These contributors could be related to only this study setting considering the policy and practices of healthcare workers. Furhter multicente cohort studies considering capturing the data on this list of factors could help to bring out associated preventable contributors. There are variabilities on neonatal care practices that could be a potential contributor requiring further analytical studies like decision on removal of a central line/umbilical catheter. There is a dearth of published literature from India requiring further research and reporting on factors contributing to neonatal HAIs.

## Conclusion

Bloodstream infection (83%) was prevalent, causing neonatal HAIs. Mechanical ventilation, NIV, and PICC line on univariate analysis contributed to neonatal HAIs. Although on univariate analysis, mechanical ventilation for > three days duration, NIV for > five days, and PICC line insertion procedure were contributors to neonatal HAIs but on multivariate analysis, NIV, PICC line, preterm labor, and LBW were found as contributors to neonatal HAIs. IV cannulation more than three times in four consecutive days was associated with neonatal HAIs; this needs further studies to find any correlation as a contributor to the neonatal HAIs.

However, mechanical ventilation, umbilical catheter, PROM, ELBW, LBW, and NBW did not pose a statistically significant risk to acquiring HAIs. A larger multicentric study from India will be required to establish further evidence.

## Data availability

### Underlying data

Figshare: Dataset on neonatal Healthcare Associated Infections of a uni-center analytical study from South India. DOI: https://doi.org/10.6084/m9.figshare.19403624 ^54^

This project contains the following underlying data:

- The data set is on the contributors to neonatal HAIs from an Indian NICU of a tertiary care teaching hospital.

Data are available under the terms of the Creative Commons Attribution 4.0 International license (CC-BY 4.0).

## Authors’ contributors

All the authors conceptualized the research idea and contributed to the manuscript. Usha Rani conceptualized, designed, analyzed, and reported the data. Leslie E. Lewis carried out technical guidance, data analysis verification, and data reporting. Usha Rani and Kiran Chawla developed a data extraction form, and later interpreted the analyzed data. Usha Rani, and Anup Naha, analyzed and reported the data. Usha Rani prepared the discussion based on the results. All the authors have proofread and has provided their intellectual contributions in the manuscript. All authors have approved the final results of the manuscript for publication.
