# Supplementary material for: Preventable contributors to the neonatal healthcare-associated infections: a uni-center analytical study from South India
Source: F1000Res. 2022 Apr 25;11:454. [Version 1] doi: 10.12688/f1000research.111101.1 (PMC9280113; doi:10.12688/f1000research.111101.1)
Supplement: Peer review history

# Peer review: Reviewer response for version 1

**DOI:** 10.5256/f1000research.122788.r143494

**Published:** 2022-07-13

**Authors:** Vijay Ivaturi

**Affiliations:** 1 Center for Translational Medicine, University of Maryland School of Pharmacy, Baltimore, MD, USA

I am pleased to review this paper titled: Preventable contributors to neonatal healthcare-associated infections: a uni-center analytical study from South India. The study focuses well on the target population, geographical location, and the preventable contributors to neonatal healthcare-associated infections. The study topic and epidemiological reporting have reported essential findings among the neonatal population considering India as one of the top countries witnessing the world's highest neonatal mortality.

 Reporting of introduction and importance of the study is adequate. The study meets the standard reporting guidelines. The methodological justification satisfies the criteria and is adequate for the inquiry. The study used easy-to-repeat, specific criteria to evaluate whether a neonate had a healthcare-associated infection. This article helps to identify the specific cases of healthcare-associated infections using diagnostic and clinical information, which is challenging. The analytical component of the article appears to have merit. The statistical tools and methods are appropriately selected and reported as per the reporting standards.

 The category of results is well-defined and meticulously planned. A novel contributor to research that was not reported earlier, like several pricks while inserting an IV cannula, was reported in this study.

 Detailed comparative discussion with global and national peer publications is adding more insight into the contributors to neonatal HAIs. Discussion is exhaustive, ensuring comparability with similar studies.

 Limitations of the study are meticulously reported and justified, which would help researchers plan future studies by overcoming these limitations.

 This study investigated the potential causes of healthcare-associated infections in newborns. Even though the study was single-center and might not be generalized to the broader population or healthcare facilities, the paper contained substantial information about preventable contributors.

 I believe that the study's outcomes will offer different perspectives to individuals who contribute to neonatal healthcare-associated illnesses. I thus suggest that this study can be considered for publication.

Is the work clearly and accurately presented and does it cite the current literature?

Yes

If applicable, is the statistical analysis and its interpretation appropriate?

Yes

Are all the source data underlying the results available to ensure full reproducibility?

Yes

Is the study design appropriate and is the work technically sound?

Yes

Are the conclusions drawn adequately supported by the results?

Yes

Are sufficient details of methods and analysis provided to allow replication by others?

Yes

Reviewer Expertise:

Predictive healthcare analytics

I confirm that I have read this submission and believe that I have an appropriate level of expertise to confirm that it is of an acceptable scientific standard.

# Peer review: Reviewer response for version 1

**DOI:** 10.5256/f1000research.122788.r137206

**Published:** 2022-05-16

**Authors:** Nitesh Kumar

**Affiliations:** 1 Department of Pharmacology and Toxicology, National Institute of Pharmaceutical Education and Research, Hajipur, Bihar, India

Dear Editorial Team,

 I am very much pleased to add my review report on the title 'Preventable contributors to the neonatal healthcare-associated infections: a uni-center analytical study from South India.' submitted by Usha Rani, Leslie E. Lewis, Kiran Chawla and Anup Naha.

 I commend the writers for selecting a topic that will benefit neonatal research in India. The paper's sequencing is well-planned. The research gap reviews are also self-evident, and the approach and instruments employed in this study offer greater relevance to the current environment. The paper's analytical section seems promising, identifying the cases of healthcare-associated infections through diagnostic and clinical evidence and later identifying the contributors in a systematic way is very challenging that is addressed in the current paper. Highlighting the contributors like frequent changes in IV cannula would guide further research in this field. Univariate and multivariate analysis of various contributors is carefully done and explained in a meaningful manner.

 They explain clearly their limitations and provide further direction and scope for research.

 The entire paper was written flawlessly in terms of language and was also educational to the entire research community. 

 The study's implications are also explicitly discussed.

 I am glad to read this paper adding value to the research community and opening doors for further research, hence, I place my recommendation for indexing the article.

Is the work clearly and accurately presented and does it cite the current literature?

Yes

If applicable, is the statistical analysis and its interpretation appropriate?

I cannot comment. A qualified statistician is required.

Are all the source data underlying the results available to ensure full reproducibility?

Yes

Is the study design appropriate and is the work technically sound?

Yes

Are the conclusions drawn adequately supported by the results?

Yes

Are sufficient details of methods and analysis provided to allow replication by others?

Yes

Reviewer Expertise:

I confirm that I have read this submission and believe that I have an appropriate level of expertise to confirm that it is of an acceptable scientific standard.

I confirm that I have read this submission and believe that I have an appropriate level of expertise to confirm that it is of an acceptable scientific standard.
